# Risk factors for death among children 0–59 months of age with moderate-to-severe diarrhea in Manhiça district, southern Mozambique

**DOI:** 10.1186/s12879-019-3948-9

**Published:** 2019-04-15

**Authors:** Sozinho Acácio, Inácio Mandomando, Tacilta Nhampossa, Llorenç Quintó, Delfino Vubil, Charfudin Sacoor, Karen Kotloff, Tamer Farag, Dilruba Nasrin, Eusébio Macete, Myron M. Levine, Pedro Alonso, Quique Bassat

**Affiliations:** 1 Centro de Investigação em Saúde de Manhiça (CISM), Rúa 12, Vila da Manhiça, 1929 Maputo, CP Mozambique; 2 Ministério da Saúde, Instituto Nacional de Saúde, Av. Eduardo Mondlane n° 1008, C.P 264 Maputo, Moçambique; 3 ISGlobal, Hospital Clínic - Universitat de Barcelona, Rosselló 132, 5-2ª, 08036 Barcelona, Spain; 4 Center for Vaccine Development (CVD), University of Maryland School of Medicine, 655 W. Baltimore Street, Baltimore, MD 21201 USA; 5 ICREA, Pg. Lluís Companys 23, 08010 Barcelona, Spain; 6 Pediatric Infectious Diseases Unit, Pediatrics Department, Hospital Sant Joan de Déu, Pg. Sant Joan de Déu 2, 08950 Barcelona, Esplugues de Llobregat Spain

**Keywords:** Moderate-to-severe diarrhea, Risk factor, Death, Children, Mozambique

## Abstract

**Background:**

Despite major improvements in child survival rates, the number of deaths due to diarrhea remains unacceptably high. We aimed to describe diarrhea-associated mortality and evaluate risk factors for death among Mozambican children with moderate-to-severe diarrhea (MSD).

**Methods:**

Between December 2007 and November 2012, children under-five with MSD were enrolled in Manhiça district, as part of the Global Enteric Multicenter study (GEMS). Clinical, epidemiological, and socio-demographic characteristics were collected. Anthropometric measurements were performed and stool samples collected upon recruitment. A follow-up visit ~ 60 days post-enrolment was conducted and verbal autopsies performed in all death cases.

**Results:**

Of the 916 MSD-cases analyzed; 90% (821/916) completed 60 days follow-up and 69 patients died. The case fatality rate at follow-up was 8% (69/821), and the mortality rate 10.2 (95%CI: 7.75–13.59) deaths per 1000 persons-week at risk. Nearly half of the deaths 48% (33/69) among study participants clustered within 2 weeks of the onset of diarrhea.

Typical enteropathogenic *Escherichia coli* (typical EPEC) and *Cryptosporidium* were the two pathogens associated to an increased risk of death in the univariate analysis with (HR = 4.16, *p* = 0.0461) and (H = 2.84, *p* = 0.0001) respectively. Conversely, *Rotavirus* infection was associated to a decreased risk of death (HR = 0.52, *p* = 0.0198)*.*

According to the multivariate analysis, risk factors for death included co-morbidities such as malnutrition (HR = 4.13, *p* <  0.0001), pneumonia/lower respiratory infection (HR = 3.51, p <  0.0001) or invasive bacterial disease (IBD) (HR = 6.80, *p* = 0.0009), presenting on arrival with lethargy or overt unconsciousness (HR = 1.73, *p* = 0.0302) or wrinkled skin (HR = 1.71, *p* = 0.0393), and cryptosporidium infection (HR = 2.14, *p* = 0.0038).

When restricting the analysis to those with available HIV results (*n* = 191, 22% of the total study sample), HIV was shown to be a significant risk factor for death (HR = 5.05, *p* = 0.0009).

Verbal autopsies were conducted in 100% of study deaths, and highlighted diarrhea as the main underlying cause of death 39%, (27/69); followed by HIV/AIDS related deaths 29.0% (20/69) and sepsis 11.6% (8/69).

**Conclusion:**

Preventive strategies targeting Cryptosporidium, malnutrition and early identification and treatment of associated co-morbidities could contribute to the prevention of the majority of diarrhea associated deaths in Mozambican children.

**Electronic supplementary material:**

The online version of this article (10.1186/s12879-019-3948-9) contains supplementary material, which is available to authorized users.

## Background

In spite of very significant decreasing trends in the last three decades, childhood mortality remains unacceptably high globally, with 5.6 million children dying every year before reaching their 5th birthday, the majority of which in low and middle-income countries (LMIC) [1]. Diarrheal diseases still represent a major cause of morbidity and mortality in childhood, and are believed to account for 499,000–526,000 annual child deaths, nearly 9% of all under five global mortality [2, 3], in spite of the good intake of life-saving interventions such as oral rehydration solution (ORS) [3] and the rotavirus vaccine [4]. Deaths in sub-Saharan Africa and Southeast Asia account for ~ 78% of deaths due to diarrhea worldwide [5, 6], underscoring the inequities related to this particular syndrome.

The Global Enteric Multi-center Study (GEMS) reported that most episodes of moderate-to-severe diarrhea (MSD) among children under 5 years old were primarily due to four pathogens: Rotavirus, Cryptosporidium, Enterotoxigenic Escherichia coli (ST-ETEC), and Shigella [7]. The risk of dying from diarrheal disease was reported to be higher among children younger than 2 years of age, albeit with relatively different rates from one region to another [7]. The advent of the HIV/AIDS pandemic has also changed the incidence [8], clinical presentation and outcome of diarrheal diseases, as the immunosuppression derived from the infection favors a higher incidence of gastrointestinal infections, not only from “classical” diarrhea pathogens, but also from more aggressive opportunistic infections, typical of the immunocompromised host. This has resulted in significant changes in the last decades in the spectrum, clinical presentation, duration and prognosis of diarrheal episodes in those countries where HIV is highly prevalent [9, 10].

Malnutrition is another public health problem in children in LMIC, often associated with an increased risk of mortality and commonly a cause and a consequence of diarrheal disease. Indeed, diarrhea episodes among malnourished children seem to be more complicated and prolonged than among non-malnourished children, and convey a worse prognosis [11–13].

In Mozambique, a paradigmatic example of a resource-constrained sub-Saharan African country, it has been estimated that diarrheal diseases are the third leading cause of death among the pediatric population (0–14 years), accounting for one out of every 10 deaths in this age group [14]. Data from the Manhiça district, in the Southern part of the country, confirm diarrheal diseases as the third leading cause of hospital admission among children 0–14 years and the fourth leading cause of death among children 12–59 months of age [15–18]. Significantly, in this same district, both HIV infection and malnutrition appear to be major public health problems, with HIV prevalence rates among adults at the community level peaking at 40% [19], and malnutrition (of any degree) affecting 47% of all children seen as outpatients [11].

Despite the significant diarrheal mortality burden, few studies have assessed the specific risk factors associated to a fatal outcome in LMIC during the diarrheal episodes. We propose in this analysis, to determine the possible risk factors associated to death among children aged 0–59 months presenting with MSD in Manhiça, a rural district in southern Mozambique with a high prevalence of infectious diseases.

## Methods

### Location of the study

The Manhiça Health Research Centre (CISM) coordinated this study, conducted in six health units in the District of Manhiça, southern Mozambique, including five peripheral health posts and the Manhiça District hospital, which acts as the referral hospital for the entire Manhiça District. This district, which is part of the wider Maputo province, is about 80 km north of Maputo, the countries’ capital. CISM established there in the year 1996 a research institution, which since then has been running in the district different research platforms, including a demographic surveillance system (DSS), covering when the study was conducted (2007–2012) an estimated population of around 92,000 inhabitants, equaling about half of the district’s total population [20]. Previous reports have detailed the idiosyncrasies of the area [21].

In parallel to the DSS, CISM also established a morbidity surveillance platform at the Manhiça District Hospital (MDH) and other surrounding smaller health posts. Such platform was designed to passively capture all pediatric (< 15 years of age) outpatient visits and all ward hospitalizations [22]. A permanent identification number (“perm-ID”), unique to each individual in the study area, and issued by the DSS, allows the round-the-clock linkage between the demographic and morbidity platforms. [23, 24].

The MDH is a busy district hospital which sees around 3500 pediatric admissions annually, and which receives over 50,000 outpatient visits. It is a 110-bed hospital, which dedicates 36 of its beds to child hospitalizations. It also includes a malnutrition specialized hospitalization unit. The MDH is supported in terms of staffing and resources by CISM. The morbidity platform uses standardized questionnaires for routine data collection, which collect demographic, clinical, laboratory and outcome data. Anthropometric measures collected include weight for all patients, and height/length among hospitalized patients. A basic microbiology surveillance protocol has also been ongoing since the year 2003, whereby for all admitted children under the age of 24 months, a blood culture is collected. For older children, this is also done in case of hyperpyrexia (documented temperature > 39°c), or based on the presence of severity criteria, or to the admitting clinician’s judgement [25, 26].

### Study population and clinical procedures

The Global Enteric Multicenter Study (GEMS) of diarrheal disease in infants and young children was conducted in seven diverse, high-burden sites in Asia and Africa, including Manhiça, in southern Mozambique. In Manhiça, GEMS recruited patients during a five-year period (Dec. 2007 to Nov. 2012). GEMS was a case-control study, stratified by age. It aimed to unravel the epidemiology and causes of pediatric diarrheal disease globally and at each of the participating sites*.* Three age groups were targeted by the study: infants (0- < 12 months), toddlers (12- < 24 months), and older children (24–- < 60 months). During the 5 years during which the study was ongoing, study staff screened for diarrhea any child under the age of five registered in the center’s DSS, who actively attended any of the participating health units. The definition for a “diarrhea episode” included the occurrence of a minimum of three loose stools in the preceding twenty-four hours.

Diarrheal episodes needed to fulfil a set of inclusion criteria to be eligible for enrolment as cases, namely that the episode was new (starting at least after a minimum of seven days in which the child had not had diarrhea), acute (shorter than a week since its beginning), or showed any of the following signs of severity, so as to fulfil the definition of “moderate-to-severe diarrhea” (MSD):

- Variables associated with dehydration: sunken eyes, decrease in skin turgor (defined by a long [≤2 s] or very long [> 2 s] abdominal skin pinch recoil), perceived need or confirmed administration of parenteral re-hydration,
- dysentery (stools with discernable blood); or
- hospitalization because of either diarrhea or dysentery.

As this was a case-control study, 1 to 3 healthy children (with no story of diarrhea in the preceding 7 days) were randomly selected using the DSS databases from the Manhiça district matched to the index case by neighborhood, age and gender. Controls were recruited no later than 2 weeks after the recruitment of each index case.

The study recorded for all enrolled individuals a variety of clinical (including anthropometric), epidemiological, and socio-demographic information. Fecal specimens were obtained for pathogen screening and characterization, following previously published methodologies [7]. As Manhiça is a highly HIV-endemic, all children were actively screened for HIV infection, after appropriate HIV counseling. This was however only started in May 2010, when the study had already recruited about a half of the required patients and continued for all study participants subsequently. Screening procedures, which followed Mozambican recommendations, utilized a two-step approach, which included first a serological screening with the Determine HIV-1/2 Rapid Test (Abbott Laboratories, Abbott Park, IL), with confirmation for all initially positive cases with a second serological test (Uni-Gold Rapid Test; Trinity Biotech Co, Wicklow, Ireland). Molecular confirmation with DNA PCR was required to confirm HIV positivity in those children under the age of 18 months with positive HIV serology. This confirmation was not required for children above the age of 18 months which were positive with serology.

The GEMS protocol included a follow-up visit at the families’ household for each enrolled participant, approximately two months after recruitment (acceptable range: 49–90 days), with the objective of verifying the health status and outcome of the diarrheal episode. Up to a maximum of 3 different contacts were attempted to trace the children who could not be found during their 60-day visit, after which, if still not found, they were considered losses to follow-up [7, 27].

In the first three months after study recruitment, any death occurring among study participants was investigated using the verbal autopsy methodology. The INDEPTH-modified standard WHO VA questionnaire was utilized. All VA questionnaires were individually reviewed by two clinicians, and discrepant diagnoses were adjudicated by an expert cause of death reviewer (QB). Of note, each clinician could attribute up to a maximum of two plausible causes of death. The final CoD diagnosis was reached when there was agreement in either one or both of the diagnoses between the two reviewer’s and/or the adjudicator. Cases in which at least two of the reviewing clinicians considered the CoD diagnosis to be “unknown” were considered undetermined. When two different final diagnoses were assigned for a given death, each of these was individually mapped onto ICD-10 in order to calculate cause-specific rates.

### Data management and statistical analysis

All data were collected using specific questionnaires, which were reviewed locally prior to being scanned and transferred to a centralized Data Collection Centre (DCC), in the US. Queries were issued centrally, but resolved locally, based on the revision of the original source documents stored on site. Stata/SE software (version 14.0, [28] and the package coxphf from R version 3.1.1 were utilized for data analyses [29, 30]. In order to estimate measures of disease burden such as diarrhea-associated mortality Rates in children, population weights based on sampling weights were calculated.

Mortality analyses were performed for MSD cases. Any death occurring during the period from recruitment to the day 60 follow-up visit was considered a study death. Time to death (or censoring) was calculated as the time span between the beginning of the diarrhea episode (first day of diarrhea, defined as the date of recruitment minus the duration of diarrhea at that moment) and the date of death (or the 60-day visit). Kaplan-Meier survival curves were estimated, and the difference between age groups was assessed by weighted-Cox regression estimation. Mortality Rates and appropriate centiles of survival time were also described (centiles in accordance to estimated survival curves).

Cox regression with Firth’s Penalized Likelihood adjusted for age [31, 32] was used to estimate the associations between the different covariates (including clinical characteristics, nutrition practices, breastfeeding and pathogens detected) and time-to-death. Multivariable models were estimated by a forward-stepwise procedure, with p-to-enter and p-to-leave both equal to 0.05 according to the Wald test. A specific multivariate analysis was conducted as a sensitivity analysis only among children with HIV results available.

In order to get a better understanding of the total community burden of diarrhea, Health Care Utilization and Attitudes Surveys (HUAS) were repeatedly conducted during the study period to investigate the what was in the area the proportion of cases usually seeking care at the different health units in the seven days following the onset of the MSD case (called r). Such surveys were conducted on random samples of children, twice a year, coinciding with each round of the DSS. Data from such surveys were pooled and weighted using the sampling weights (inverse of sampling fraction), based on the number of children in each age-sex stratum according to information derived from CISM’s DSS at the time of the round [7]. After that, the values of “r” and its variances were estimated for each age stratum, using Kaplan Meier analysis.

A weighted analysis, as previously described by Sommerfelt et al [33], was utilized for the extrapolation of mortality rates to the population levels. Such method proposes to choose weights so as to make the proportion of cases in the analysis similar to that of the population from which the cases are drawn. For the calculation of weights applicable to controls, the inverse of sampling fraction within each age stratum was used, according to the formula: *(N-cases in_pop)/controls*, where *N* is the median population; *cases_in pop* is the number of cases in the population estimated by the number of eligible cases divided by r and *controls* implies the number of enrolled controls for that stratum. Weights for cases were constructed according to the sampling weights adjusted for fortnight quote (described above) divided by r.

### Ethical considerations

This study, which was conducted under the GEMS framework, was coordinated by the Center for Vaccine Development (Baltimore, Maryland, USA) and led by CISM in Manhiça, in close collaboration and partnership with the Barcelona institute of Global Health (ISGlobal), in Spain. All GEMS-related protocols and informed consent documents were reviewed and approved prior to the initiation of the study by the National Bioethics Committee for Health of Mozambique **(CNBS – IRB00002657)**, the ethics committee of the Hospital Clínic of Barcelona, and The Institutional Review Board at the University of Maryland. All parents/guardians of participating children were requested to sign a written informed consent after adequate information regarding the study was provided.

## Results

**Fig. 1:**
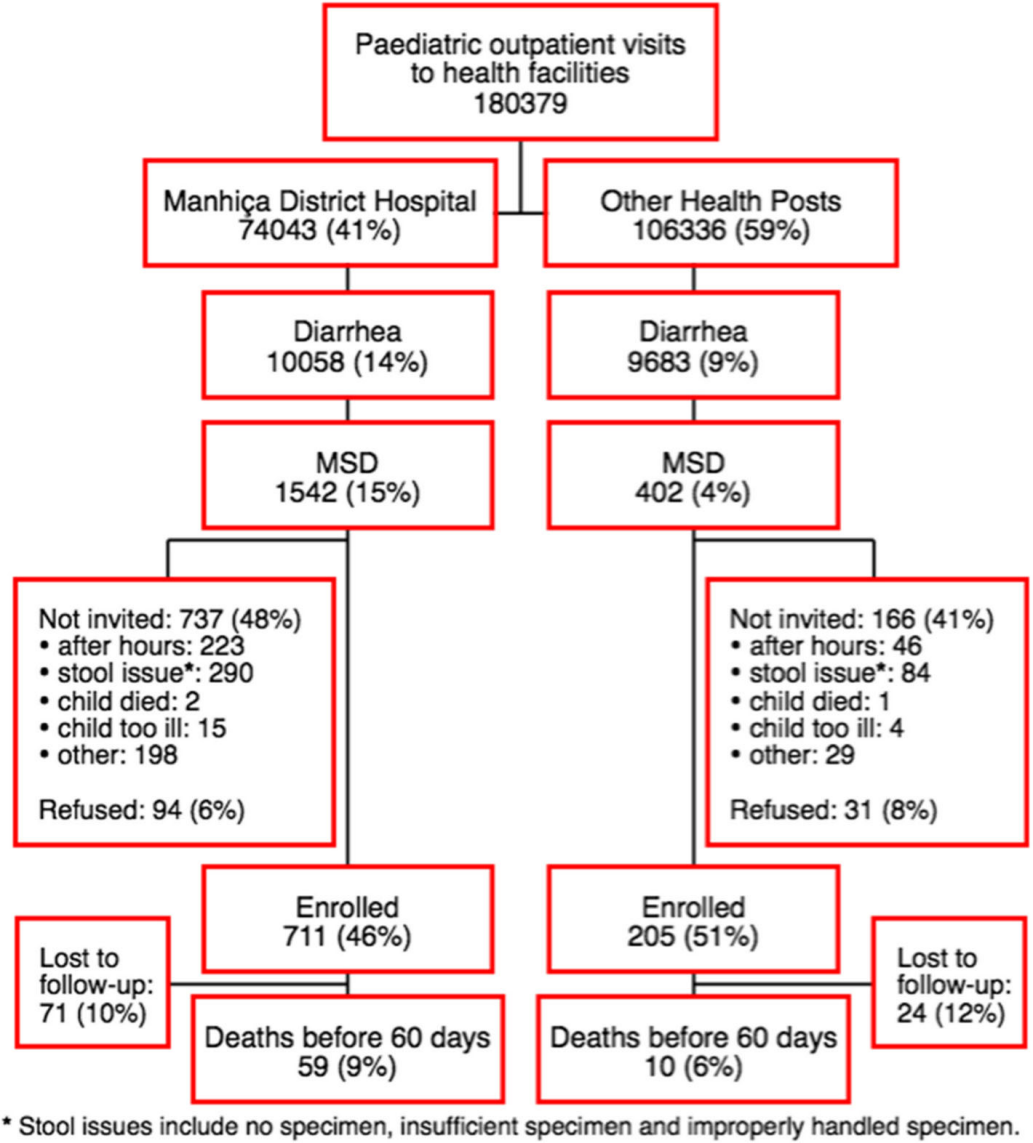
Hospital visits due to moderate-to-severe diarrhea among children aged 0–59 months of age within DSS area in Manhiça, Mozambique, December 2007 – November 2012

The study included data on childhood deaths among children recruited in Manhiça with MSD, between December 2007 and November 2012 as part of the GEMS. We enrolled a total of 916 children with MSD and 90% (821/916) of them completed the day 60 follow-up home visit (Fig. 1). Reasons for not completing the 60-day follow-up were essentially due to either changes of residence to other areas outside the DSS area, or temporary absences from their homes coinciding with the 60-day follow-up visits. In these cases, and according to the study protocol, children were declared lost to follow-up if 3 consecutive visits were conducted after day 60 with no success in finding the child.

As HIV counseling and testing was only introduced for study participants in May 2010, HIV data are missing for many recruited participants (100% for year 1 and 2; 73% for year 3, 36% for year 4 and 16% for year 5). However, no relevant differences were shown when comparing children with or without HIV available data (data not shown).

**Fig. 2:**
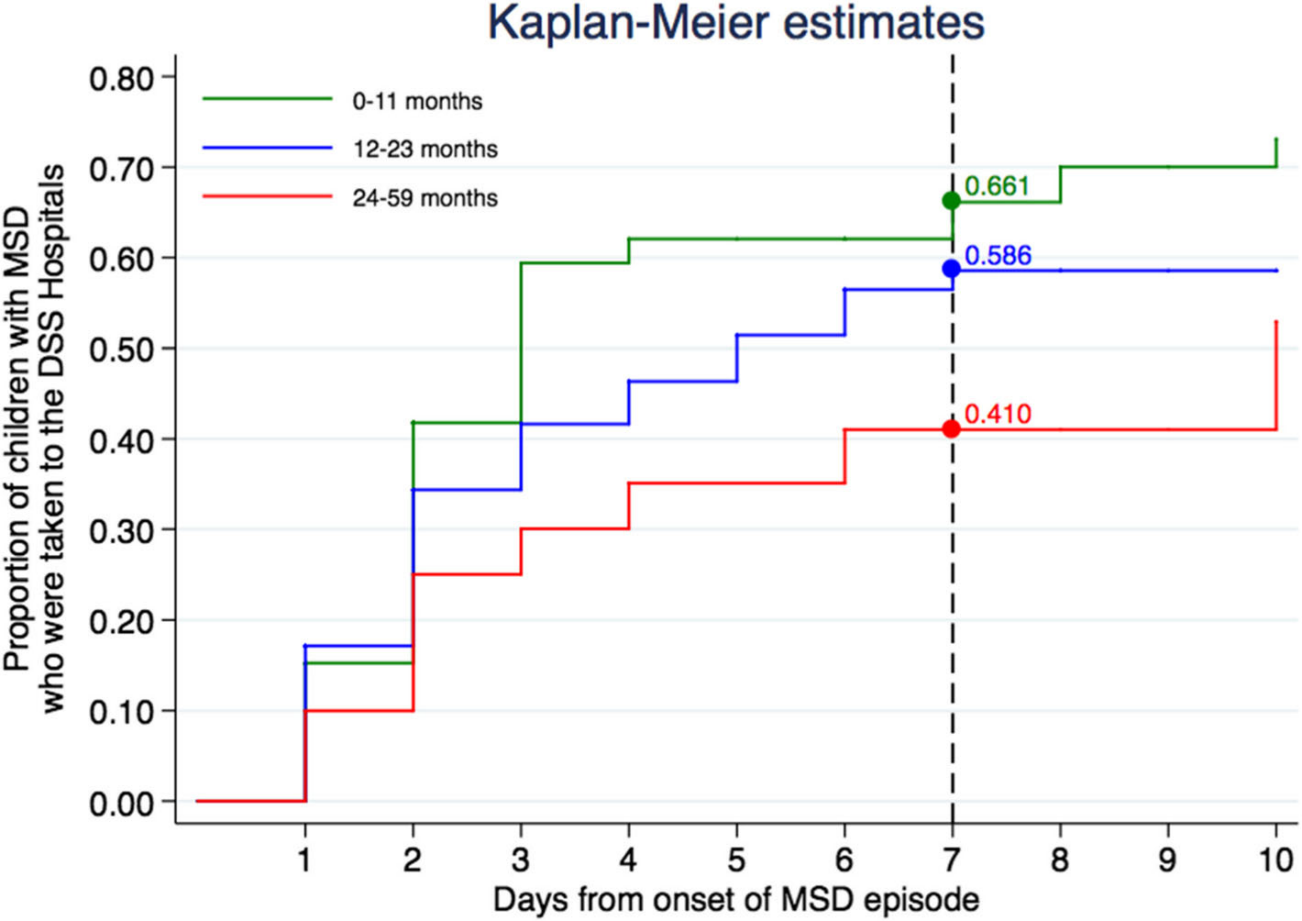
Proportion of children who sought health care within 7 days of onset of episode

The proportion of children with MSD who were taken to the hospital within the first 7 days from onset of episode was 66, 59 and 41% among infants, toddlers and children, respectively **(**Fig. 2).

**Fig. 3:**
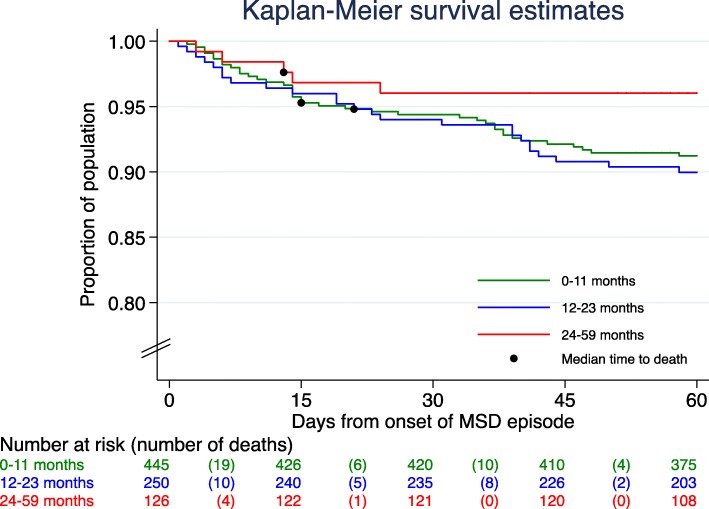
Mortality at 60 days follow-up among children 0–59 months of age with diarrhea with estimated median time to death for each age group

Sixty-nine deaths were detected among study participants during the study follow-up (recruitment-day up to last follow-up visit), providing a case fatality rate of 8% (69/821). The mortality rate after adequately weighting was 10.2 (95%CI: 7.75–13.59) deaths per 1000 persons-week at risk (PWAR). According to the age group the mortality rate was 10.9 (95%CI: 7.71–15.90) deaths per 1000 PWAR among infants, 11.9 (95%CI: 7.82–18.81) deaths per 1000 PWAR among toddlers and 5.9 (95%CI: 1.85–28.55) deaths per 1000 PWAR among children 24–59 months of age (*p* = 0.5357) **(**Fig. 3 and Table 1). No differences were found in terms of the distribution of deaths in each age group in relation to time, with 48% (33/69) of all deaths occurring within the first two weeks after the onset of the diarrhea episode, of which 25% (17/69) in the first 7 days, and 23% (16/69) after the first week and before completing 14 days of onset. In terms of distribution of deaths according to sex, no significant differences were observed [deaths among boys 57% (39/69) compared to 43% (30/69) among girls; hazard ratio of death [HR = 1.12 (95%CI: 0.79–1.80, *p* = 0.6199)].

**Table 1:** Mortality at 60 days follow-up among children 0–59 months of age with MSD

Age	Unweighted estimates		Weighted estimates									
	Subjects	Deaths	Subjects	Deaths	Time at risk (pwar)^a^	Mortality Rate			Survival time centiles			
						Estimation per 1000 pwar	(95% CI)	p-value^*^	1%	5%	25%	50%
0–11 months	445	39 (9%)	1218	121 (10%)	11,097.01	10.91	(7.71, 15.90)	reference	4	14	.	.
12–23 months	250	25 (10%)	931	100 (11%)	8429.69	11.87	(7.82, 18.81)	0.7914	3	21	.	.
24–59 months	126	5 (4%)	570	32 (6%)	5345.62	5.96	(1.85, 28.55)	0.3115	3	14	.	.
OVERALL	821	69 (8%)	2719	253 (9%)	24,872.33	10.17	(7.75, 13.59)	–	3	17	.	.

^*^Weighted Cox regression

^a^Person-weeks at risk

### Microbiologic profile

Pathogens associated with an increased risk of death in the univariate analysis were *Cryptosporidium* (HR = 2.84 (95%CI: 1.72–4.58, *p* = 0.0001) and typical EPEC (HR = 4.16 (95%CI: 1.02–38.05, *p* = 0.0461), with Astrovirus infection (HR = 3.51, 95%CI: 0.97–8.93, *p* = 0.0545) bordering statistical significance. Conversely, cases infected with Rotavirus were less likely to die due to diarrhea (HR = 0.52, 95%CI: 0.38–0.90, *p* = 0.0198) (Table 2).

**Table 2:** Pathogens associated with death due to moderate-to-severe diarrhea in the univariate analysis, in children 0–59 months of age

Variable	Vital child’s status at 60-day visit		HR (95%CI)	*p*-value
	Alive *n* = 752 (%)	Dead *n* = 69 (%)		
ETEC (any ST)	46/172 (27%)	5/14 (36%)	1.64 (0.52, 4.66)	0.3730
ETEC (LT only)	1/643 (0%)	0/57 (0%)	6.17 (0.04,.)	0.3228
EAEC (aata only)	109/752 (14%)	10/69 (14%)	0.97 (0.47, 1.80)	0.9291
EAEC (aaic only)	64/752 (9%)	4/69 (6%)	0.73 (0.24, 1.71)	0.5107
EAEC (aata/aaic)	37/752 (5%)	1/69 (1%)	0.42 (0.04, 1.53)	0.2242
typical EPEC	45/71 (63%)	14/15 (93%)	4.16 (1.02, 38.05)	0.0461
atypical EPEC	1/178 (1%)	0/20 (0%)	4.28 (0.03, 31.01)	0.4095
Shigella (any)	43/752 (6%)	3/69 (4%)	1.01 (0.27, 2.69)	0.9785
Rotavirus	251/751 (33%)	15/69 (22%)	0.52 (0.285, 0.905)	0.0198
Aeromonas spp	9/752 (1%)	0/69 (0%)	0.63 (0.005,.)	0.7282
Norovirus (all)	30/751 (4%)	1/69 (1%)	0.54 (0.06, 1.99)	0.4207
Adenovirus (40/41)	16/749 (2%)	0/68 (0%)	0.34 (0.003,.)	0.3625
Adenovirus (non 40/41)	10/749 (1%)	1/68 (1%)	1.43 (0.16, 5.45)	0.6483
V. cholerae 01	12/752 (2%)	1/69 (1%)	1.94 (0.21, 8.00)	0.4810
Non-typhoidal Salmonella	7/752 (1%)	0/69 (0%)	0.72 (0.006,.)	0.8107
Cryptosporidium	121/751 (16%)	26/69 (38%)	2.84 (1.72, 4.58)	0.0001
Giardia	133/751 (18%)	10/ 69 (14%)	0.90 (0.433, 1.70)	0.7590
Entamoeba histolytica	75/750 (10%)	9/69 (13%)	1.42 (0.67, 2.69)	0.3292
Campylobacter (all)	35/752 (5%)	2/69 (3%)	0.72 (0.15, 2.09)	0.5978
Astrovirus	10/751 (1%)	3/69 (4%)	3.51 (0.97, 8.93)	0.0545
Sapovirus	12/751 (2%)	0/69 (0%)	0.42 (0.003,.)	0.4870
Helicobacter pylori	28/115 (24%)	0/8 (0%)	0.21 (0.002, 1.79)	0.1894
Ascaris lumbricoides	9/115 (8%)	0/8 (0%)	0.79 (0.006, 7.02)	0.8713
Strongyloides stercoralis	4/115 (3%)	0/8 (0%)	2.06 (0.01, 22.34)	0.6617
Ancylostoma duodenale	2/115 (2%)	0/8 (0%)	9.42 (0.04, 326.81)	0.3067
Necator americanus	2/115 (2%)	0/8 (0%)	9.42 (0.04, 326.81)	0.3067

### Clinical characteristics

Univariate associations between clinical characteristics and time-to-death were also assessed (Table 3). Admission to hospital implied a higher risk of death (HR 3.53, 95%CI 1.95–7.52, *p* <  0.0001). The median time of hospitalization was significantly longer for those who died (6.5 days) than for those who survived (4.0 days, p <  0.0001). Clinical variables on arrival associated to mortality included, among others, lethargy or impaired consciousness, fever, signs of dehydration such as loss of skin turgor and skin pinch, chest indrawing and breathing difficulties, signs associated to malnutrition (“flaky paint” skin appearance, abnormal hair) and rectal prolapse. Only a small fraction of recruited children 22% (191/821) had available HIV results, and positivity rate among these was high 21% (40/191). Among these, HIV infection was strongly correlated with the risk of death (HR = 6.04, 95%CI 2.38–16.09, *p* = 0.0002). Invasive bacterial disease (bacteremia in an admitted child) (HR = 17.44, 95%CI 6.43–38.15, *p* <  0.0001) and pneumonia/lower respiratory infections (HR = 3.51, 95%CI 2.16–5.65, p <  0.0001) diagnosed during hospital admission were co-morbidities strongly associated with a lethal outcome. Malnourished children had also a significantly increased risk of dying (HR = 5.66, 95%CI 3.43–9.14, *p* <  0.0001)*.* After adjusting for anthropometric characteristics at enrollment, breastfed children had a lower mortality risk (HR = 0.28, 95%CI 0.13–0.69 for partially breastfeed children; HR = 0.27, 95%CI 0.11–0.64 for exclusively breastfed children; *p* = 0.0036)]. Conversely, an admission diagnosis of dysentery was associated with a 70% reduction in the risk of dying (HR = 0.30, 95%CI 0.08–0.78, *p* = 0.0112).

**Table 3:** Clinical characteristics associated with death due to moderate-to-severe diarrhea in the univariate analysis, in children 0–59 months of age

Variable		Vital child’s status at 60-day visit		Total	Hazar Ratio	*p*-value
		Alive	Dead			
Demographic characteristics						
Gender: Female^a^		307 / 752 (41%)	30 / 69 (43%)	337 / 821 (41%)	1.127 (0.698, 1.804)	0.6199^*^
Clinical history before hospital admission						
Oralite or ors^a^		202 / 752 (27%)	24 / 69 (35%)	226 / 821 (28%)	1.404 (0.846, 2.274)	0.1840^*^
Special milk or infant formula^a^		1 / 752 (0%)	0 / 69 (0%)	1 / 821 (0%)	5.277 (0.042,.)	0.3565^*^
Home remedy/herbal medication^a^		138 / 752 (18%)	19 / 69 (28%)	157 / 821 (19%)	1.617 (0.936, 2.684)	0.0832^*^
No special remedies given^a^		399 / 752 (53%)	32 / 69 (46%)	431 / 821 (52%)	0.800 (0.497, 1.281)	0.3533^*^
How long has this episode lasted^b^		2.00 (2.00) [752]	2.00 (2.00) [69]	2.00 (2.00) [821]	1.117 (0.922, 1.330)^c^	0.2473^*^
Describe stool^a^	Simple watery	422 (56%)	48 (70%)	470 (57%)	reference	0.0630^*^
	Rice watery stool	12 (2%)	0 (0%)	12 (1%)	0.404 (0.003,.)	
	Sticky/Mucoid	200 (27%)	18 (26%)	218 (27%)	0.815 (0.465, 1.368)	
	Bloody	118 (16%)	3 (4%)	121 (15%)	0.281 (0.076, 0.747)	
	Total	752 (100%)	69 (100%)	821 (100%)		
Hospital admission						
Child was admitted to hospital^a^		471 / 752 (63%)	60 / 69 (87%)	531 / 821 (65%)	3.533 (1.858, 7.520)	< 0.0001*
Received antibiotics^a^		413 / 752 (55%)	56 / 69 (81%)	469 / 821 (57%)	3.179 (1.799, 6.035)	< 0.0001*
Number of days admitted^b^		4.00 (3.00) [471]	6.50 (7.00) [58]	4.00 (2.00) [529]	1.103 (1.069, 1.132)^c^	< 0.0001*
Signs/symptoms any time since beginning of episode						
Vomiting 3 or more times per day^a^		363 / 752 (48%)	33 / 69 (48%)	396 / 821 (48%)	0.942 (0.585, 1.512)	0.8037^*^
Unable to drink^a^		61 / 752 (8%)	5 / 69 (7%)	66 / 821 (8%)	0.993 (0.367, 2.165)	0.9875*
Fever^a^		249 / 751 (33%)	31 / 69 (45%)	280 / 820 (34%)	1.620 (1.006, 2.591)	0.0474^*^
Belly pain^a^		188 / 752 (25%)	19 / 69 (28%)	207 / 821 (25%)	1.162 (0.673, 1.926)	0.5784^*^
Loss of consciousness^a^		9 / 752 (1%)	3 / 69 (4%)	12 / 821 (1%)	4.554 (1.258, 11.556)	0.0248^*^
Wrinkled skin^a^		195 / 751 (26%)	33 / 69 (48%)	228 / 820 (28%)	2.436 (1.513, 3.912)	0.0003^*^
Irritable or restless^a^		105 / 752 (14%)	8 / 69 (12%)	113 / 821 (14%)	0.812 (0.367, 1.577)	0.5600*
Lethargy or loss of consciousness^a^		344 / 750 (46%)	44 / 69 (64%)	388 / 819 (47%)	1.989 (1.233, 3.279)	0.0047^*^
Convulsion^a^		29 / 752 (4%)	1 / 69 (1%)	30 / 821 (4%)	0.632 (0.071, 2.335)	0.5514^*^
Difficulty breathing^a^		53 / 751 (7%)	14 / 69 (20%)	67 / 820 (8%)	3.182 (1.705, 5.578)	0.0006^*^
Chest indrawing^a^		113 / 747 (15%)	18 / 68 (26%)	131 / 815 (16%)	1.948 (1.110, 3.276)	0.0213^*^
Fast breathing^a^		171 / 750 (23%)	19 / 69 (28%)	190 / 819 (23%)	1.218 (0.696, 2.055)	0.4784^*^
Rectal prolapse^a^		1 / 751 (0%)	2 / 69 (3%)	3 / 820 (0%)	12.096 (2.501, 35.288)	0.0053^*^
Dehydration and nutritional status						
Very thirsty^a^		570 / 750 (76%)	48 / 69 (70%)	618 / 819 (75%)	0.754 (0.459, 1.282)	0.2890^*^
Drinks poorly^a^		72 / 751 (10%)	11 / 68 (16%)	83 / 819 (10%)	1.776 (0.894, 3.226)	0.0967^*^
Sunken eyes^a^		368 / 751 (49%)	38 / 69 (55%)	406 / 820 (50%)	1.231 (0.769, 1.985)	0.3861^*^
Loss of skin turgor^a^		208 / 752 (28%)	33 / 69 (48%)	241 / 821 (29%)	2.234 (1.388, 3.586)	0.0011^*^
Dry mouth^a^		298 / 751 (40%)	37 / 69 (54%)	335 / 820 (41%)	1.680 (1.050, 2.703)	0.0306^*^
I ntravenous rehydration^a^		345 / 752 (46%)	39 / 69 (57%)	384 / 821 (47%)	1.441 (0.895, 2.342)	0.1325^*^
Skin pinch^a^	Normal	529 (70%)	36 (52%)	565 (69%)	reference	0.0020^*^
	Slow return (<= 2 s.)	172 (23%)	21 (30%)	193 (24%)	1.690 (0.974, 2.864)	
	Very slow (> 2 s.)	51 (7%)	12 (17%)	63 (8%)	3.356 (1.696, 6.203)	
	Total	752 (100%)	69 (100%)	821 (100%)		
Dehydration (at least one of above)^a^		698 / 752 (93%)	66 / 69 (96%)	764 / 821 (93%)	1.393 (0.549, 5.042)	0.5256^*^
Length/height-for-age z-score (median height)^d^		−1.35 (1.38) [746]	−2.27 (1.59) [68]	−1.43 (1.42) [814]	0.648 (0.554, 0.760)^c^	< 0.0001^*^
Bipedal edema^a^		11 / 752 (1%)	3 / 69 (4%)	14 / 821 (2%)	3.237 (0.891, 8.271)	0.0701^*^
Abnormal hair^a^		57 / 752 (8%)	18 / 69 (26%)	75 / 821 (9%)	3.827 (2.193, 6.388)	< 0.0001*
Skin has ‘flaky paint’ appearance^a^		9 / 752 (1%)	4 / 69 (6%)	13 / 821 (2%)	4.602 (1.509, 10.721)	0.0108*
Is the child currently breastfed^a^	No	150 (24%)	21 (35%)	171 (25%)	reference	0.0036^*^
	Partial breastfeeding	297 (47%)	23 (38%)	320 (46%)	0.281 (0.134, 0.597)	
	Exclusive breastfeeding	190 (30%)	16 (27%)	206 (30%)	0.275 (0.117, 0.641)	
	Total	637 (100%)	60 (100%)	697 (100%)		
Hospital diagnoses/co-morbidities						
Dysentery^a^		120 / 752 (16%)	3 / 69 (4%)	123 / 821 (15%)	0.302 (0.082, 0.786)	0.0112^*^
Pneumonia/lower resp. infection^a^		125 / 752 (17%)	30 / 69 (43%)	155 / 821 (19%)	3.510 (2.162, 5.651)	< 0.0001^*^
Meningitis^a^		0 / 752 (0%)	1 / 69 (1%)	1 / 821 (0%)	62.693 (6.820, 258.067)	0.0023^*^
Other invasive bacterial infection^a^		1 / 752 (0%)	5 / 69 (7%)	6 / 821 (1%)	17.447 (6.431, 38.158)	< 0.0001^*^
Malaria^a^		129 / 752 (17%)	10 / 69 (14%)	139 / 821 (17%)	0.878 (0.430, 1.619)	0.6920^*^
Malnutrition^a^		63 / 752 (8%)	26 / 69 (38%)	89 / 821 (11%)	5.658 (3.439, 9.140)	< 0.0001^*^
HIV^a^	Tested negative	144 (19%)	7 (10%)	151 (18%)	reference	0.0019*
	Tested positive	30 (4%)	10 (14%)	40 (5%)	6.041 (2.384, 16.090)	
	Untested pre-May2010	507 (67%)	48 (70%)	555 (68%)	1.789 (0.882, 4.200)	
	Untested from May2010 onwards	71 (9%)	4 (6%)	75 (9%)	1.155 (0.327, 3.629)	
	Total	752 (100%)	69 (100%)	821 (100%)		

^*^Cox Regression with Firth’s Penalized Likelihood adjusted for age and CI based on the Profile Penalized-Log Likelihood

^a^n (Column percentage)

^b^Median (IQR) [n]

^c^Per unit increase

^d^Arithmetic Mean (SD) [n]

### Multivariate analysis

Independent risk factors for death in the multivariate analysis included co-morbidities such as malnutrition (HR = 4.13, 95%CI: 2.42–6.96; *p* <  0.0001), pneumonia/lower respiratory infection (HR = 3.50, 95%CI 2.15–5.67, p <  0.0001) or invasive bacterial disease (IBD) (HR = 6.80, 95%CI 2.43–15.50, *p* = 0.0009), presenting on arrival with lethargy or overt unconsciousness (HR = 1.73, 95%CI 1.05–2.90, *p* = 0.0302) or wrinkled skin (HR = 1.71, 95%CI 1.02–2.84, *p* = 0.0393), and a diagnosis of cryptosporidium infection (HR = 2.14, 95%CI 1.28–3.58, *p* = 0.0038) (Table 4).

**Table 4:** Independent risk factors associated to death in cases with Moderate-to-severe diarrhea (MSD (Multivariate analysis)

Variable	Hazard Ratio	95% Conf. Interval	*p*-value^(*)^
Age (months)^(a)^	0.995	(0.963, 1.023)	0.7433
Malnutrition	4.132	(2.428, 6.877)	< 0.0001
Pneumonia/lower resp. infection	3.515	(2.158, 5.682)	< 0.0001
Invasive bacterial disease (IBD)	6.804	(2.435, 15.516)	0.0009
Cryptosporidium	2.142	(1.289, 3.487)	0.0038
Lethargy or loss of consciousness	1.730	(1.054, 2.903)	0.0300
Wrinkled skin	1.712	(1.027, 2.846)	0.0395

^*^Cox Regression with Firth’s Penalized Likelihood adjusted for age and CI based on the Profile Penalized-Log Likelihood

^a^Hazard Ratio is the relative change in Hazard per one month increase in age

### Verbal autopsy cause of death diagnoses

Verbal autopsies were conducted to all 69 deaths documented in study participants (Additional file 1: Table S1). Final cause of death (CoD) ascertained by this method highlighted diarrhea as the main underlying CoD 39% (27/69), followed by HIV/AIDS related deaths 29% (20/69) and sepsis 12% (8/69).

## Discussion

The unprecedented decreases in child mortality that the world has experienced in the last years is the result of multiple factors, including significant reductions in certain major causes of under 5 mortality, such as childhood diarrhea. Indeed, the last three decades have seen diarrhea deaths dropping down from the 1.748 million deaths in the year 1990 [34], to the estimated half a million deaths in 2015 [1]. However, understanding that good preventive and curative strategies are now widely implemented, diarrhea associated mortality remains unacceptably high. This study has highlighted the associated impact of diarrheal disease in Manhiça, a rural area of Southern Mozambique, whereby up to 8.4% of the children recruited during a 5-year long period died within the first 2 months after the episode, yielding a population weighted mortality rate of 10.2 deaths/1000 persons-week at risk. The fact that nearly half of all deaths clustered within the first two weeks after the initiation of the diarrhea episode is strongly supportive of the significance of this pathology starting the chain of events leading to death, rather than other causes. This is also supported by the cause of death diagnoses provided by the verbal autopsies conducted among all study deaths, whereby 39% of all deaths were directly attributed to diarrhea. More work is therefore needed to put in place better preventive and management tools to specifically address diarrhea as a syndrome, and also all its secondary consequences.

This study allows a better understanding of the risk factors and associated problems that require more attention in order to address diarrheal mortality in this particular setting. Independent risk factors for death identified through this analysis are multifactorial and can be subdivided in those specifically related to pathogens, clinical signs and symptoms, or to concurrent co-morbidities.

Cryptosporidium was the only pathogen independently associated with death, even after adjusting for covariates. This pathogen had already been identified in the GEMS study as one of the major culprits of excess mortality among young children with MSD, being Manhiça the GEMS site where the highest overall prevalence was found [35], whereby cryptosporidium represented the second most frequent pathogens associated with MSD [18]. Infection from this protozoan, that can affect both immunocompetent but also immunocompromised hosts, often derives from contact with potentially contaminated water or food sources. In the absence of a Cryptosporidium vaccine and considering the ineffectiveness of current treatment strategies in the youngest populations and the diagnostic challenges posed by this pathogen, minimizing the overall environmental burden (contaminated water/food) needs prioritization. Additionally, and given the high prevalence of HIV infection in the district of Manhiça [19], prompt initiation of antiretroviral therapy among those children confirmed to be HIV-infected may contribute to a reduction in the burden of Cryptosporidium infections, understanding that this is another well characterized risk factor for cryptosporidiosis [36].

The presence of an *Astrovirus* infection appeared as an independent risk factor for death in the multivariate analysis restricted to children with available HIV data, the association being borderline significant (HR 3.5, *p* = 0.0545) in the univariate analysis of the entire population. Although numbers are relatively small, more attention should be drawn to this particular pathogen, that has already demonstrated a substantial disease burden in neighboring countries [37], and for which neither curative nor preventive strategies have been described.

Although rotavirus-associated mortality has clearly been established globally [38], in this series rotavirus infections appeared to protect against death in the univariate analysis (HR = 0.5, *p* = 0.0198), highlighting overall good course secondary to this pathogen if adequate hydration measures are promptly started at the community of health system level.

Being admitted to hospital appeared as a risk factor for a poorer outcome. This is hardly surprising, considering that those patients with MSD requiring admission are those with higher severity, and with a major risk of co-morbidities [39], and that health care utilization in cases of MSD in this study was high. In terms of signs and symptoms detected in patients upon admission associated with a higher risk of death, those related to an altered consciousness and dehydration were most relevant, together with those suggestive of respiratory distress and/or nutritional deficiencies. These signs/symptoms reflect both the real mechanisms by which a diarrheal episode can become lethal and the typical complications of MSD. Indeed, guaranteeing an adequate intake of liquids and replacing excessively lost water and electrolytes during episodes of diarrhea remains the cornerstone strategy to prevent diarrheal associated deaths, and was at the basis of the WHO and UNICEF’s recommendation of promoting Oral Rehydration Solution (ORS) usage back in the 1970’s [40]. However, diarrheal episodes can trigger both secondary infections or the exacerbation of pre-existing conditions, such as malnutrition, as historically and extensively demonstrated in the literature [41] and also as confirmed in this study, where malnutrition was one of the strongest risk factors associated with death. Breaking the vicious circle that can become established between malnutrition and diarrheal diseases will therefore be critical to address a significant proportion of all MSD deaths in settings such as Manhiça.

Breast-feeding, especially if this is the only source of nutrition, has been shown to protect children against diarrhea in Africa as elsewhere in the developing world [42]. Previous analyses from this same study showed that in Manhiça, when controlled by other confounders, the particular breastfeeding pattern did not seem to protect against pediatric MSD [18] . Interestingly, and in accordance with the published evidence [43], this analysis has shown that breastfeeding (either partial or exclusive) during the first months of life has a significant protective effect against diarrheal attributable mortality. Our findings reinforce the current WHO recommendations that encourage exclusive breastfeeding during the first 6 months of life as a vital strategy to prevent infant mortality [44, 45].

A concomitant diagnosis of pneumonia (HR = 3.50, *P* <  0.0001) or invasive bacterial disease (HR = 6.79, *P* = 0.0009), were both clear and independent risk factors for death, signaling the importance of co-morbidities as triggers of a fatal outcome, and of concomitant antibiotic treatment as a potential justifiable strategy in patients with MSD which do not improve rapidly with adequate rehydration. Importantly, an admission diagnosis of dysentery was shown in this analysis to be protective against death in the univariate analysis (HR = 0.3, *P* = 0.0112). Typical pathogens causing dysentery such as Shigella or *Entamoeba histolytica* have been shown to cause longer and more severe diarrheal episodes, with an overall increased risk of associated mortality [46, 47]. Our somehow counterintuitively finding may be explained by the early and aggressive administration of antimicrobials in cases of diarrhea with blood in our setting, which may have attenuated the impact of this particular type of diarrheal disease.

In this series, MSD episodes among HIV-infected individuals carried a much worse prognosis. Although not even a fourth of all patients had HIV screening results available, due to the late initiation of this practice during the study, the prevalence of HIV-infected individuals among recruited patients was high (21%), and this infection was clearly recognized as an independent risk factor in the multivariate analysis restricted to those with HIV data available. Indeed, HIV infection has been shown to play a significant role not only in the burden and associated mortality of MSD, but also in the etiology and epidemiology of diarrheal disease among children in Manhiça [48]. In this respect, in settings such as Manhiça with a high HIV prevalence, aggressive and prompt initiation of antiretrovirals among newly diagnosed HIV cases should be the pillar upon which basing any strategy destined to reducing the burden of diarrheal deaths. Similarly, the suitability of exclusive breastfeeding practices for this particular vulnerable group should also be strengthened [49].

### Limitations

This study has several limitations, starting with the aforementioned lack of HIV data for all study participants, which would have allowed a more precise characterization of the risk factors for MSD mortality. This happened because according to the national guidelines, HIV testing was not mandatory and required an additional patient consent; leading to the high proportion of participants without HIV results. However, the comparison of patients with or without HIV results available showed no differences (data not shown). This study failed to evaluate electrolyte or zinc levels among MSD cases and controls, which could have contributed to a better understanding of dehydration patterns or to the enrichment of the evidence supporting zinc supplementation in children with diarrhea. However, several previous studies have demonstrated the protective effect of this simple intervention against diarrhea mortality [50, 51] and its use remains warranted, something that was not in place in Mozambique during the study period. In addition, there was also a potential for recall bias during the administration of verbal autopsy forms in this study. Causes of death based on verbal autopsies were determined by physicians, although this method also has inherent limitations for its subjectivity and low reliability as described elsewere [52–55]. Finally, this analysis was conducted with GEM’s original set of microbiological data, and more recent re-analyses of the study samples, using more advanced molecular methods, have evidenced a different distribution of the pathogens associated to MSD, something that could have potentially altered our own analysis.

## Conclusions

By characterizing diarrhea-associated deaths in a large group of Mozambican children, this study contributed to enlarging the pool of evidence regarding risk factors for mortality among pediatric patients with MSD. The knowledge of these factors can better define those children at higher risk of dying as a result of a diarrheal episode, and thus allow a more targeted and specific approach to prevent those deaths. In this setting, targeted measures against Cryptosporidium infection and aggressive management of dehydration, malnutrition and suspected co-morbidities, together with the promotion of continuous breastfeeding may help prevent most deaths due to diarrhea.

## Additional file

Additional file 1: **Table S1.** Main cause of death diagnoses among study participants as determined by verbal autopsy. (DOCX 14 kb)

## Abbreviations

atypical EPEC: atypical enteropathogenic Escherichia coli
CoD: Cause of death
DCC: Data Collection Centre in the United States of America
DSS: Demographic Surveillance System
EAEC: Enteroaggregative Escherichia coli
GEMS: Global Enteric Multi-center Study
INDEPTH: The International Network for the Demographic Evaluation of Populations and Their Health
LMIC: Low and middle-income countries
LSD: Less-severe diarrhea
LT–ETEC: Enterotoxigenic Escherichia coli producing heat-labile toxin
MSD: Moderate-to-severe diarrhea
ORS: Oral rehydration solution
ST–ETEC: Enterotoxigenic Escherichia coli producing heat-stable toxin
typical EPEC: Typical enteropathogenic Escherichia coli
VA: Verbal autopsy
