# Supplementary material for: Risk factors for death among children 0–59 months of age with moderate-to-severe diarrhea in Manhiça district, southern Mozambique
Source: BMC Infect Dis. 2019 Apr 15;19:322. doi: 10.1186/s12879-019-3948-9 (PMC6466733; doi:10.1186/s12879-019-3948-9)
Supplement: Supplementary file 1 — Table S1. Main cause of death diagnoses among study participants as determined by verbal autopsy. (DOCX 14 kb) [file 12879_2019_3948_MOESM1_ESM.docx]

**Supplementary** **Table 1** Main cause of death diagnoses among study participants as determined by verbal autopsy

|  | **Deaths (n=69)** |
| --- | --- |
| **Causes of death (according to VA) as primary or co-primary diagnosis (ICD-10 codes)*** |  |
| Diarrheal diseases (A09) | 27/69 (39.1%) |
| Acute resp. Infection, including pneumonia (J18) | 6/69 (8.7%) |
| HIV/AIDS related death (B24) | 20/69 (29.0%) |
| Severe malnutrition (E46) | 3/69 (4.3%) |
| Sepsis (including neonatal) (A41 and P36) | 8/69 (11.6%) |
| Malaria (B54) | 4/69 (5.8%) |
| *As each patient may have up to 2 different diagnoses, numbers are not additive to 100% | |
